# Supplementary material for: Scaling Up the Process of Titanium Dioxide Nanotube Synthesis and Its Effect on Photoelectrochemical Properties
Source: Materials (Basel). 2021 Sep 29;14(19):5686. doi: 10.3390/ma14195686 (PMC8510213; doi:10.3390/ma14195686)
Supplement: Supplementary file 1 [file materials-14-05686-s001.zip › materials-1380780-supplementary.pdf]

Supplementary Information

Scaling-up of the process of titanium dioxide nanotubes synthesis and its effect on photoelectrochemical properties

Mariusz Szkoda [1,2]*, Konrad Trzciński [1],Zuzanna Zarach [1], Daria Roda [1], Marcin Łapiński [2,3] and Andrzej P. Nowak [1]

[1] Faculty of Chemistry, Department of Chemistry and Technology of Functional Materials, Gdańsk University of Technology, Narutowicza 11/12, 80-233, Gdańsk, Poland
[2] Advanced Materials Center, Gdańsk University of Technology, ul. Narutowicza 11/12, 80-233, Gdańsk, Poland
[3] Advanced Materials Center, Gdańsk University of Technology, ul. Narutowicza 11/12, 80-233, Gdańsk, Poland
* Correspondence: mariusz-szkoda1@pg.edu.pl; Tel.: +48 58 348 64 34 (MS)

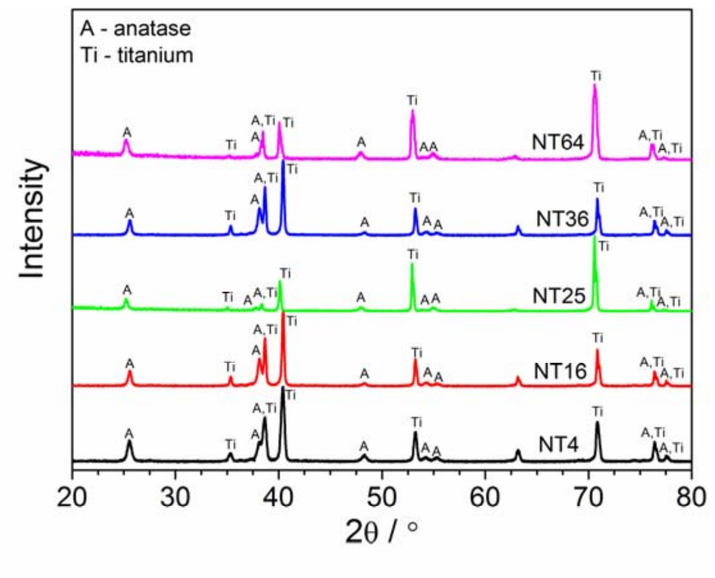

Figure S1. Comparison of XRD patterns of TiO_2 samples.

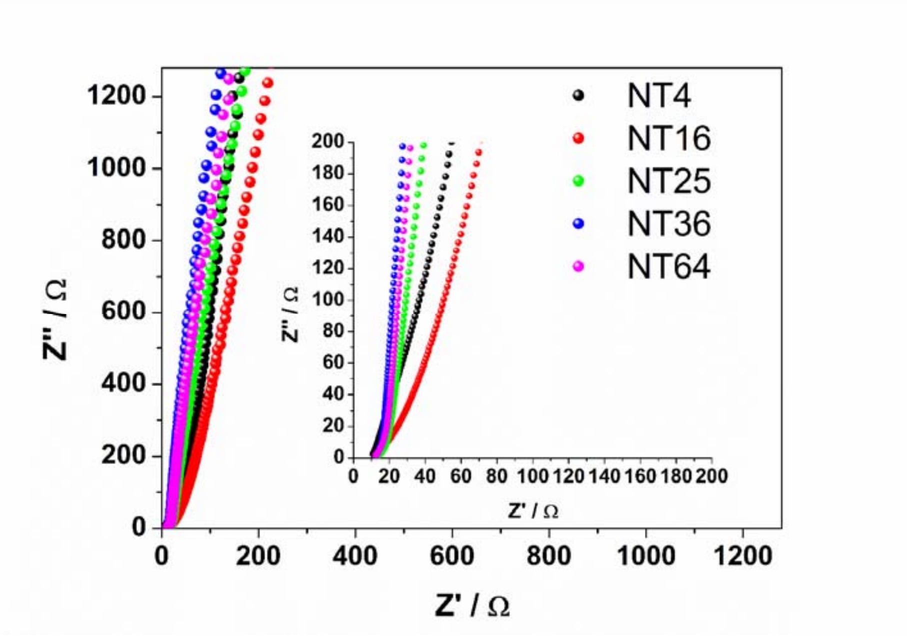

Figure. S2. EIS spectra of the analyzed samples, performed in 0.2M K_2SO_4.

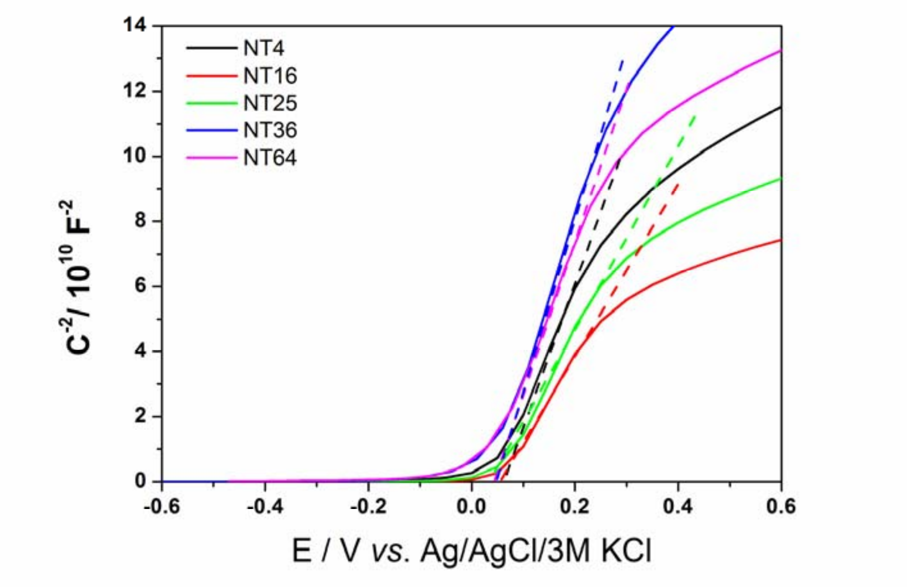

Figure. S3. The Mott-Schottky plot for obtained electrodes.
